# A scalable system for generation of mesenchymal stem cells derived from induced pluripotent cells employing bioreactors and degradable microcarriers

**DOI:** 10.1002/sctm.21-0151

**Published:** 2021-09-10

**Authors:** Robert E. Rogers, Andrew Haskell, Berkley P. White, Sujata Dalal, Megan Lopez, Daniel Tahan, Simin Pan, Gagandeep Kaur, Hyemee Kim, Heather Barreda, Susan L. Woodard, Oscar R. Benavides, Jing Dai, Qingguo Zhao, Kristen C. Maitland, Arum Han, Zivko L. Nikolov, Fei Liu, Ryang Hwa Lee, Carl A. Gregory, Roland Kaunas

**Affiliations:** ^1^ Department of Molecular and Cellular Medicine, Texas A&M Health Science Center College of Medicine Bryan Texas USA; ^2^ Department of Biomedical Engineering Texas A&M University, Emerging Technologies Building College Station Texas USA; ^3^ National Center for Therapeutics Manufacturing Texas A&M University College Station Texas USA; ^4^ Department of Electrical and Computer Engineering Texas A&M University, Wisenbaker Engineering Building College Station Texas USA; ^5^ Biological and Agricultural Engineering Texas A&M University, Scoates Hall College Station Texas USA

**Keywords:** bioreactor, immunomodulatory, induced pluripotent stem cells, mesenchymal stem cells

## Abstract

Human mesenchymal stem cells (hMSCs) are effective in treating disorders resulting from an inflammatory or heightened immune response. The hMSCs derived from induced pluripotent stem cells (ihMSCs) share the characteristics of tissue derived hMSCs but lack challenges associated with limited tissue sources and donor variation. To meet the expected future demand for ihMSCs, there is a need to develop scalable methods for their production at clinical yields while retaining immunomodulatory efficacy. Herein, we describe a platform for the scalable expansion and rapid harvest of ihMSCs with robust immunomodulatory activity using degradable gelatin methacryloyl (GelMA) microcarriers. GelMA microcarriers were rapidly and reproducibly fabricated using a custom microfluidic step emulsification device at relatively low cost. Using vertical wheel bioreactors, 8.8 to 16.3‐fold expansion of ihMSCs was achieved over 8 days. Complete recovery by 5‐minute digestion of the microcarriers with standard cell dissociation reagents resulted in >95% viability. The ihMSCs matched or exceeded immunomodulatory potential in vitro when compared with ihMSCs expanded on monolayers. This is the first description of a robust, scalable, and cost‐effective method for generation of immunomodulatory ihMSCs, representing a significant contribution to their translational potential.

## Graphical abstract

A scalable system for the generation and rapid harvest of immunomodulatory induced pluripotent stem cell‐derived mesenchymal stem cells..

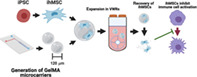

**Significance statement**

Human mesenchymal stem cells are effective in treating disorders resulting from an inflammatory or heightened immune response. In the present study, the authors use a single inexhaustible source of human mesenchymal stem cells generated from induced pluripotent stem cells. The authors report procedures for manufacture of the cells in scalable bioreactors while attached to novel digestible microcarriers that permit rapid harvest at high yields and viability. This is the first description of a robust, scalable, and cost‐effective method for generation of immunomodulatory human mesenchymal stem cells derived from induced pluripotent cells, representing a significant contribution to the translational potential of the cells.

## 1 INTRODUCTION

Human mesenchymal stem cells (hMSCs) and their secreted products have been found to be effective in treating disorders resulting from a heightened immune response, demonstrated by several clinical trials and preclinical studies.^1^, ^2^, ^3^ In particular, hMSCs and hMSC‐derived exosomes have been used successfully to treat refractory graft‐vs‐host disease, rheumatoid arthritis, and perianal fistulas of Crohn's disease in humans.^3^, ^4^, ^5^, ^6^, ^7^ To meet the expected future demand for hMSCs, there is a pressing need to develop scalable methods for production of hMSCs at clinically relevant yields while retaining robust immunomodulatory function.^8^, ^9^, ^10^

Current methods for production of hMSCs are largely reliant on monolayer culture techniques. However, hMSC expansion on microcarriers in bioreactors is a more efficient, scalable, and cost‐effective approach to produce the billions of cells necessary for therapeutic lot production.^11^, ^12^, ^13^ Microcarrier expansion can be achieved without compromising the general characteristics of hMSCs or their therapeutic potency, and the process has even been found to improve effectiveness in immunomodulatory applications.^14^, ^15^

Commercially available microcarriers have diverse characteristics that impact the ability to expand and recover hMSCs.^16^, ^17^ Polystyrene‐based microcarriers have the advantage of robust mechanical properties, are easily sterilized, and have been approved for the generation of clinically compatible cell products, but they are nondegradable, requiring filtration to remove the hMSCs following expansion. In many cases, these purification processes can affect yields and viability resulting in significant losses due to clogging, shear stress and kinetic damage.^18^, ^19^ Degradable microcarriers, such as gelatin‐based microcarriers, improve the recovery of cells by circumventing the need for filtration.^20^ However, gelatin‐based commercially available microcarriers are porous, facilitating invasion of the hMSCs into the microcarrier. This can result in apoptosis of the cells at the core of these microcarriers and can adversely affect yields and viability.^21^

Combining a nonporous, biodegradable microcarrier with scalable bioreactor technologies could provide a cost‐effective platform for improved manufacture of hMSCs and their related products. Gelatin methacryloyl (GelMA) is a photocrosslinkable derivative of gelatin that permits integrin‐binding and enzymatic‐degradation.^22^, ^23^ Microcarriers prepared from GelMA have previously been used for the expansion of hMSCs on low attachment well plates without penetration of hMSCs into the microcarrier.^23^ However, GelMA microcarriers have not yet been used for the expansion of hMSCs in scalable bioreactors for immunomodulatory applications.

For robust manufacture of hMSCs at clinically relevant yields, it is necessary to not only optimize bioreactor and microcarrier platforms, but also to select the appropriate cellular origin for MSC expansion. Induced pluripotent stem cell (iPSC)‐derived hMSCs (ihMSCs) can be prepared from a single line of iPSCs, permitting the production of trillions of cells with very reproducible properties from a single, renewable source.^24^, ^25^ The immunomodulatory capabilities of ihMSCs are comparable to those from bone marrow‐derived hMSCs both in vitro and in preclinical models of inflammatory diseases.^26^ However, the expansion and recovery of immunoregulatory ihMSCs using scalable bioreactor technologies has not yet been reported.

In this study, we hypothesized that GelMA microcarriers produced using a step emulsification microfluidic device can serve as a degradable microcarrier platform for the expansion of ihMSCs in scalable bioreactors without compromising standard MSC characteristics or immunomodulatory capabilities. To test this hypothesis, ihMSCs were expanded on GelMA microcarriers in vertical wheel bioreactors (VWBs) or rotating wall vessel bioreactors (RWVBs) and evaluated based on their standard MSC properties and immunomodulatory potential. We compared the results of our fabricated GelMA microcarriers with monolayer culture and a commercially available collagen‐coated polystyrene microcarrier (Pall‐Solohill) that has previously been used for hMSC expansion in bioreactors.^16^, ^27^, ^28^, ^29^

## 2 METHODS

A diagrammatic summary of the experimental plan is provided in Supporting Information Figure S1.

### 2.1 GelMA synthesis

GelMA was synthesized after modifications were made to a protocol by Loessner et al.^30^ Briefly, type A porcine gelatin, Bloom 300 (Sigma) was reacted with methacrylic anhydride (0.6 mL methacrylic anhydride per gram gelatin) in phosphate‐buffered saline (PBS) for 1 hour at 50°C with vigorous stirring. After quenching the reaction with 40°C PBS and dialyzing for 7 days through a 12 to 14 kDa nitrocellulose membrane against deionized water at 40°C, the pH was readjusted to 7.4 using 1 M NaHCO_3_, filtered through a 0.2 μm polyether‐sulphone filter, and lyophilized for 7 days in 0.2 μm filtered conical tubes. The number of functionalized amines was determined using a fluorescamine reagent according to the manufacturer's protocol (Thermo Fisher Scientific). In total, eight batches of GelMA microcarriers were synthesized from different batches of gelatin. The source material or GelMA batch did not significantly affect size or cytocompatibility. Some batches of GelMA microcarriers were subjected to E‐beam sterilization at the National Center for Electron Beam Research, (Texas A&M University, College Station, Texas) utilizing a 10 MeV/18 kW S‐Band microwave‐based linear accelerator delivering a total of 30 kGy.

### 2.2 Microfluidic device production

A master mold for the microfluidic device was developed on a silicon wafer with SU‐8 2025 (20 μm height for nozzle channel) and 2075 photoresist (180 μm height for dispensing and continuous phase channel, Microchem, Inc) using conventional soft photolithography.^31^ Polydimethylsiloxane (PDMS, 10:1 mixture, Sylgard 184) was poured over the master mold and polymerized at 85°C for 1 hour. PDMS components and a 2″ × 3″ glass slide were exposed to 1 minute of oxygen plasma treatment, pressed together to form a covalent bond, and cured overnight at 85°C. The assembled microfluidic device was then treated with Aquapel (3M) to render the microfluidic surface hydrophobic and sterilized by autoclaving prior to microcarrier production.

### 2.3 Microcarrier production

GelMA and photoinitiator lithium phenyl‐2,4,6‐trimethylbenzoylphosphinate were dissolved in PBS at 50°C to final concentrations of 4% or 7.5% (w/v) and 10 mM, respectively. The GelMA solution and fluorinated oil (Novec 7500 with 1% [v/v] PicoSurf‐1, Sphere Fluidics) were added to syringes and connected to the microfluidic device using Tygon ND 100‐80 tubing (0.01″ ID × 0.03″ OD, Saint Gobain). Syringe pumps (Cole Parmer) were used to perfuse the device with GelMA and fluorinated oil streams at 2 and 4 mL per hour, respectively, for droplet generation. Droplets and fluorinated oil flowed into Tygon ND 100‐80 tubing (0.02″ ID × 0.06″ OD, Saint Gobain) and were exposed to 365 nm‐wavelength ultraviolet light (75 mW per cm^2^) for 50 seconds to polymerize the microcarriers before collection into a 50 mL conical tube. After removing the majority of fluorinated oil by pipet, the microcarriers were separated from the residual oil by centrifugation over a 20 mL glycerol bed at 3000*g* for 60 seconds. Each 20 mL glycerol extraction was sufficient to generate microcarriers for 10 100‐mL VWB cultures. Microcarriers were transferred to another 50 mL conical tube through a 300 μm strainer, washed three times using PBS, and stored at 4°C until use. Microcarrier diameters were quantified using bright‐field microscopy during and after purification using a custom MATLAB (Mathworks) code. Histograms and plots of diameters during production were generated using Prism (GraphPad).

### 2.4 Measuring compressive modulus

Compressive moduli of 4% and 7.5% GelMA were measured using bulk hydrogels. Briefly, 200 μL 4% and 7.5% GelMA prepolymer solutions with LAP in PBS were crosslinked in 8 mm disk molds and allowed to swell in PBS at room temperature for 15 hours. Compressive modulus was measured via dynamic mechanical analysis at 37°C using a stress‐strain sweep at a rate of 5% strain/minute for 3 minutes (Q800, TA Instruments). Average compressive modulus was determined during 5% to 10% strain on three independent hydrogels at each GelMA concentration.

### 2.5 Monolayer cell culture

Induced pluripotent stem cell‐derived human mesenchymal stem cells were provided by Dr. Fei Liu (Texas A&M College of Medicine) and prepared as previously described.^24^ Cells were seeded at 500 cells per cm^2^ on tissue culture polystyrene and expanded in complete culture medium (CCM) containing α‐minimum essential media (Invitrogen), 10% (v/v) fetal bovine serum (FBS, R&D Systems, Atlanta Biologicals), 4 mM L‐glutamine, and 100 U/mL penicillin and streptomycin changed every 2 to 3 days. Upon reaching 70% confluence (about 15 000 cells per cm^2^, six population doublings), cells were exposed to 0.25% trypsin, 0.1% EDTA (Corning) at 37°C for 5 minutes. After deactivating trypsin with CCM, cells were collected by centrifugation at 500*g* for 5 minutes. One cycle of seeding and recovery was defined as a single expansion passage and ihMSCs from passage 3 to 6 were used for experiments.

### 2.6 Attachment experiments

To determine the attachment efficiency of the cells to microcarriers, ihMSCs were added to 25 cm^2^ (equivalent surface area) of microcarriers to attain a final seeding density of 1000, 5000, or 10 000 cells per cm^2^ in low attachment 6‐well plates (Corning). GelMA microcarriers were compared to commercially available collagen‐coated polystyrene (Pall‐Solohill) microcarriers. After adjusting the volume of each well to 5 mL with CCM, plates were agitated with a uniaxial rotor at 6 RPM (Mimetas) for 22 hours in a cell culture incubator. The cells were harvested from the microcarriers by incubation with 0.25% trypsin/0.1% EDTA for 5 minutes at 37°C, and the cells collected from the collagen‐coated polystyrene spheres were passed through a 40 μm cell strainer (PET mesh, PluriSelect). The cells were pelleted at 500*g* for 5 minutes and quantified using a hemocytometer.

To measure short term expansion on microcarriers in 5 mL plated cultures, a total of 1000 or 6600 ihMSCs per cm^2^ were seeded onto 25 cm^2^ of GelMA in low attachment 6‐well plates. Half of the media was exchanged every 2 days after collecting the microcarriers into 15 mL conical tubes and allowing them to settle. The cells were harvested on day 8 and counted using a hemocytometer.

### 2.7 Rotating wall vessel bioreactor expansion

A total of 1000 ihMSCs per cm^2^ were seeded onto 90.4 cm^2^ of GelMA or collagen‐coated polystyrene (Pall‐Solohill) microcarriers in 10 mL Rotating Wall Vessel Bioreactors (Synthecon). Cell attachment occurred over 1 hour during on/off cycles of 24 RPM for 1 minute followed by 0 RPM for 20 minutes. After this, the reactors were set for continuous operation at 24 RPM. Every 2 days, half of the media in each bioreactor was replaced with fresh CCM. Cells were expanded on microcarriers until day 6 or day 8, at which point they were washed twice with 10 mL PBS before exposure to 5 mL 0.25% trypsin/0.1% EDTA at 37°C for 5 minutes with agitation. After trypsin deactivation with CCM, the cells were collected, strained, and used for subsequent experiments.

### 2.8 Vertical wheel bioreactor expansion

A total of 1000 ihMSCs per cm^2^ were seeded onto 500 cm^2^ of GelMA or collagen‐coated polystyrene (Pall‐Solohill) microcarriers in PBS‐0.1MAG vertical wheel bioreactors (PBS Biotech) containing 100 mL CCM. The ihMSCs were allowed to attach to microcarriers for 16 hours during on/off cycles of 12 RPM for 1 minute followed by 0 RPM for 20 minutes. After 16 hours, the bioreactor speed was increased to 17 RPM and changed to continuous operation. Every 2 days, half of the media in each bioreactor was replaced with fresh CCM after allowing the microcarriers to settle. On day 4, the speed of reactors containing collagen‐coated polystyrene microcarriers was increased to 20 RPM to prevent microcarrier settling, while reactors containing GelMA microcarriers were maintained at 17 RPM as there was lack of settling. These speeds were maintained until the end of the bioreactor expansion on day 8.

To assess expansion on the microcarrier surfaces, 10 mL aliquots of the GelMA or polystyrene microcarriers were removed every 2 days for ihMSC quantification. Microcarriers were washed with PBS and cells were removed via exposure to 0.25% trypsin/0.1% EDTA for 5 minutes at 37°C with agitation. After deactivation of the trypsin with CCM, ihMSCs were separated from polystyrene microcarriers using a 40 μm cell strainer and pelleted at 500*g* for 5 minutes. Since GelMA microcarriers completely degraded during trypsin exposure, the cells did not require straining after trypsin deactivation and were directly pelleted. The number of ihMSCs was determined using a hemocytometer and divided by the microcarrier surface area to determine the degree of cell expansion. After 8 days of expansion, microcarriers were removed from the reactor and allowed to settle in 50 mL conical tubes before aspiration of liquid culture media. Microcarriers were washed twice with 50 mL of PBS before the addition of 30 mL 0.25% trypsin/0.1% EDTA and incubation for 5 minutes at 37°C under 120 RPM agitation on an Advanced Digital Shaker (VWR). After the addition of culture media to deactivate trypsin, ihMSCs were separated from collagen‐coated polystyrene microcarriers using a 40 μm cell strainer while ihMSCs isolated from GelMA microcarriers were not. Cells were collected after centrifugation at 500*g* for 5 minutes in CCM and yield/viability was determined using trypan blue exclusion visualized on a hemocytometer.

### 2.9 Imaging of microcarriers

Microcarriers were immobilized in 1% (w/v) agarose and imaging was performed on a Leica SP8 confocal microscope in reflectance mode using a 10× objective lens (HP PL APO 10X/0.40 CS2, Leica). The white light laser illumination wavelength was filtered to 638 nm, and the detection spectrum was set to 635 to 645 nm to collect scattered light. Images were acquired with 2.4 μm axial step size through a <250 μm scan depth to capture scattering from cells attached to the surface of the microspheres. Z‐projections were generated using ImageJ software (National Institutes of Health).

### 2.10 MSC functional validation assays

Colony forming capabilities of recovered cells were determined as previously described.^32^ Briefly, a total of 100 ihMSCs were seeded in 150 cm^2^ dishes with 20% (v/v) FBS‐containing CCM and incubated for 21 days before staining with 3% (w/v) crystal violet in methanol. Colonies were counted by hand to determine the percentage of cells in culture that were capable of forming single‐cell derived colonies.^33^

Osteogenic differentiation was evaluated using previously described methods.^34^, ^35^ Briefly, cells were expanded in 12‐well plates before exposure to osteogenic basal medium (OBM) consisting of 5 mM β‐glycerophosphate (Sigma), 50 μg per mL ascorbic acid (Sigma), and CCM containing 20% FBS. Media was exchanged every 2 to 3 days to promote mineralization. Note that unlike bone marrow derived MSCs, ihMSCs do not require supplemental dexamethasone to induce mineralization.^36^ After 21 days, monolayers were washed with PBS and fixed with 4% (w/v) paraformaldehyde (PFA) in PBS for 15 minutes. After fixation, monolayers were washed with PBS and deionized (DI) water before staining with 40 mM Alizarin Red S (ARS) in pH 4.0 water for 30 minutes. Residual ARS was washed away with DI water and imaged using bright field microscopy. The amount of ARS in each well was quantified using absorbance at 405 nm after dye extraction using 10% (v/v) acetic acid in water.^34^, ^37^

Adipogenic differentiation was evaluated using previously described methods.^34^ Briefly, cells expanded in 12‐well plates were exposed to adipogenic differentiation medium (ADM) consisting of 500 nM dexamethasone (Sigma), 500 nM isobutylmethylxanthine (Sigma), 500 nM indomethacin (Sigma), and CCM containing 20% FBS. Media was exchanged every 3 to 4 days to promote lipid droplet formation. After 28 days of differentiation, monolayers were fixed with 4% (w/v) PFA in PBS and washed with PBS before staining with 0.6% Oil Red‐O in 60% isopropanol/40% PBS for 30 minutes. Monolayers were washed with PBS and imaged using bright field microscopy.

### 2.11 Immunophenotype characterization

Passage 5 ihMSCs were cultured on 7.5% GelMA or collagen I‐coated polystyrene microcarriers (Pall SoloHill) in 0.1 L VWBs or on 150 cm^2^ culture plates (Corning) for 8 days. Cells were harvested, suspended in phosphate buffered saline containing 2% (v/v) fetal bovine serum (Atlanta Biologicals), and stained 1:100 with the following antibodies (Beckman Coulter): CD11b‐PE Bear1 ASR, CD14‐PE RMO52 ASR, CD19‐FITC J3‐119 ASR, CD34‐FITC 581 ASR, CD45‐FITC J33 ASR, CD73‐PE AD‐2 ASR, CD79a‐PE HM47 ASR, CD90‐PE (1:25), CD105‐PE TEA3/17.1.1 ASR, and HLA‐DR, DP, DQ‐FITC 18142 ASR. Stained cells were incubated on ice in the dark for at least 10 minutes and 10 000 events were analyzed (BD Fortessa).

### 2.12 Immunomodulatory potential

Animal studies were performed in accordance with Texas A&M Institutional Animal Care and Use Committee approved protocols. The immunomodulatory potential of isolated ihMSCs was evaluated using in vitro LPS stimulation of murine splenocytes as previously described.^15^, ^38^, ^39^ Splenocytes (5 × 10^5^ cells per well) isolated from 6‐week‐old BALB/c or C57BL/6J mice (Jackson Laboratory, Bar Harbor, ME) mice were stimulated with 50 ng per mL LPS in RPMI containing 5% heat‐inactivated FBS with 100 U/mL penicillin/100 mg/mL streptomycin. The splenocytes were mixed with ihMSCs (2.5 × 10^3^, 5 × 10^3^ and 10 × 10^3^ cells per well resulting in a ratio of 1:200, 1:100 and 1:50 splenocytes per ihMSC) plated the day before. After 24 hours, the concentration of IFN‐γ, IL‐6, TNF‐α in cell‐free supernatant was determined using ELISA in triplicate according to the manufacturer's protocol. Three wells of splenocyte culture were used as technical replicates.

### 2.13 Reverse transcriptase real‐time PCR

Assays were performed on RNA extracted from 5 × 10^5^ ihMSCs recovered from 5 days of monolayer or GelMA culture. RNA extractions and PCR assays were performed using previously reported standard protocols^24^ with primer sequences retrieved from the Primerbank database (pga.mgh.harvard.edu/primerbank).

### 2.14 Statistics

Unless otherwise stated, all statistics were performed assuming that a *P*‐value less than .05 meets conventional standards of statistical significance. Differences in attachment densities were measured using two‐way ANOVA with Tukey's multiple comparisons test. Differences in cell densities in low‐attachment plates and VWBs were determined using two‐tailed *t*‐tests. Differences in expansion between the two microcarrier cultures were evaluated using a repeated measures ANOVA, and differences between cell densities on each day were determined using Sidak's multiple comparison test. Difference in cell viability was determined using two‐tailed *t*‐tests. Differences in ARS intensity were measured using a one‐way ANOVA with Tukey's multiple comparisons test. Differences in cytokine secretion in the murine splenocyte assay were determined using a one‐way ANOVA with Dunnet's multiple comparisons test.

## 3 RESULTS

### 3.1 GelMA microcarrier production using step emulsification

**FIGURE 1:**
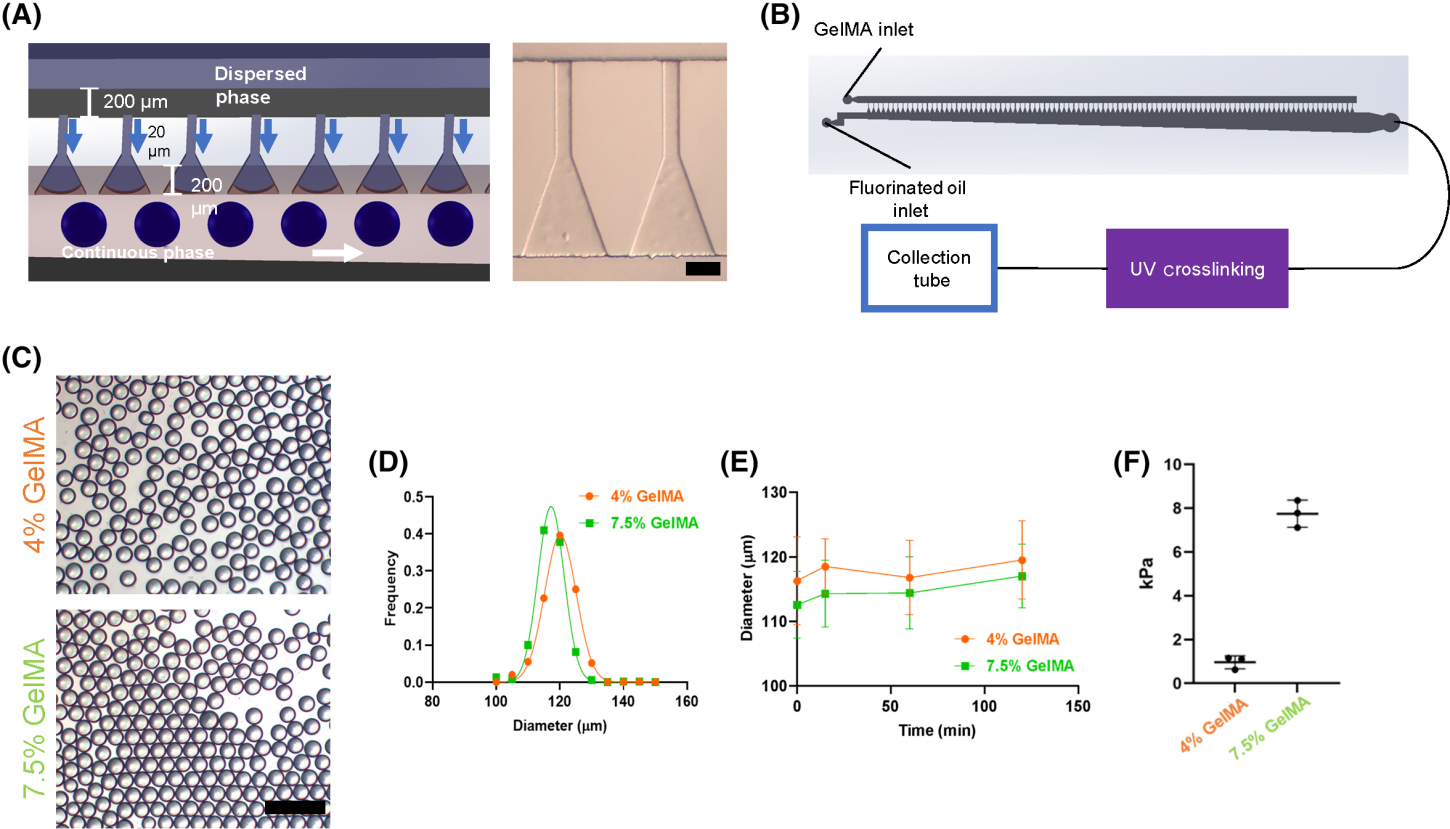
Uniform GelMA droplets are generated using step emulsification microfluidics. A, A schematic of the microfluidic device (left) demonstrates the dimensions and the flow pattern of the GelMA (dispersed) phase and the fluorinated oil (continuous) phase. A micrograph (right) of two units within the device (scale bar = 100 μm). B, A diagram of the microcarrier production process indicates the location of UV crosslinking unit between droplet generation and the collection tube. Not shown in this diagram is that the GelMA, fluorinated oil, and microfluidic device were maintained in a 40°C heated environment. C, Bright field images of 4% and 7.5% GelMA microcarriers demonstrate uniform diameter distributions after crosslinking and collection (scale bar = 500 μm). D, Microcarriers prepared from 4% and 7.5% GelMA adopt normal diameter distributions at approximately 118 to 120 μm with an appreciable degree of overlap between the two microcarrier preparations. E, Average and variance of microcarrier diameters during 2 hours of production. Data presented in this figure are representative of three independent microcarrier productions. F, Compressive moduli of 4% and 7.5% GelMA

A 100‐channel step emulsification microfluidic device was fabricated to rapidly (3 × 10^6^ microcarriers per hour) generate monodisperse microcarriers of reproducible size (Figure 1A,B, Supporting Information S2).^40^ Microcarriers produced from both 4% and 7.5% (w/v) GelMA had uniform diameters of approximately 120 μm that followed normal distributions with coefficients of variation less than 5% (Figure 1C,D). In addition, the diameter of both 4% and 7.5% GelMA microcarriers was stable over the course of a 120‐minute run, with the average diameters always within one SD of one another (Figure 1E). For initial batches, E‐beam sterilization was performed without detriment to the shape and size of the microcarriers or their ability to expand ihMSCs. Compressive moduli of 4% and 7.5% GelMA materials were 1 and 7.9 kPa, respectively (Figure 1F).

### 3.2 ihMSC expansion on GelMA microcarriers

**FIGURE 2:**
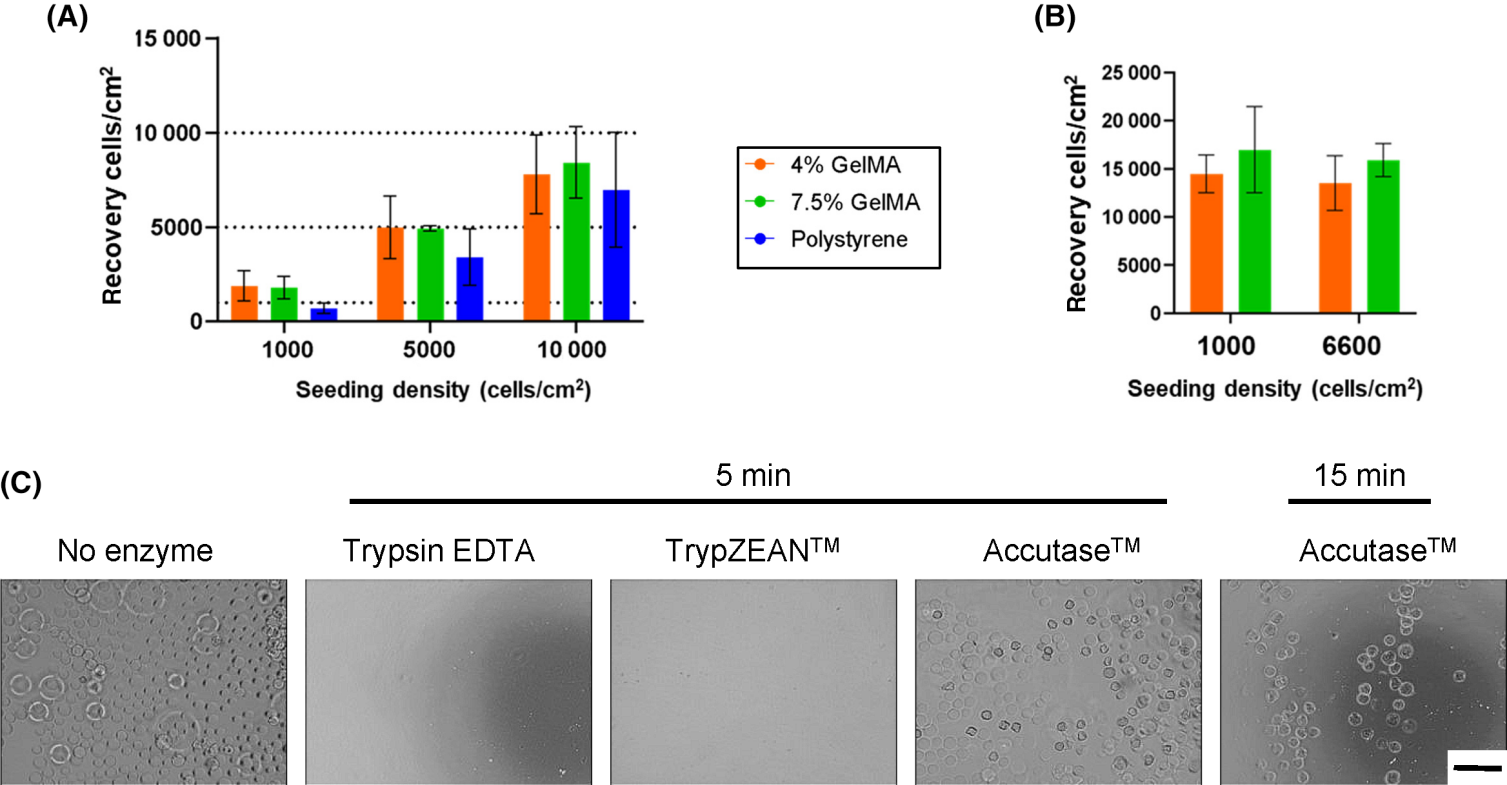
GelMA microcarriers facilitate efficient attachment, support ihMSC expansion, and are rapidly degraded using Trypsin and TrypZEAN: A, The number of ihMSCs recovered after 22 hours of attachment yielded similar quantities to the initial number added. Horizontal dashed lines were added indicating the original seeding densities. B, Expansion of ihMSCs over 8 days on 4% and 7.5% GelMA microcarriers in shallow plated cultures. C, Representative images of GelMA microcarriers after incubation with the indicated protease at 37°C for the indicated duration (scale bar = 500 μm)

A range of different ihMSC seeding densities were tested in pilot experiments performed in plated microcarrier cultures. For this purpose, microcarriers with a total surface area of 25 cm^2^ per experiment were suspended with ihMSCs seeded at 1000, 5000 and 10 000 cells per cm^2^ in 6 mL media, in standard low‐attachment 9 cm^2^ plates. For comparison, a commonly utilized commercial microcarrier consisting of collagen I coated polystyrene microcarriers (hereafter referred to as polystyrene) was also assayed in the same manner. After 22 hours with gentle agitation, the cells seeded at 1000 and 5000 cells/cm^2^ on GelMA microcarriers exhibited approximately 100% attachment and recovery, whereas recovery from polystyrene microcarriers was consistently lower at approximately 80% recovery (Figure 2A). With seeding at 10000 cells/cm^2^, attachment and recovery was approximately 80% of the input in all cases and statistical significance was not observed with several repetitions of the experiment (Figure 2A). When the ihMSCs were seeded at 1000 and 6600 cells per cm^2^ (a seeding density similar to those used in other studies^12^) and incubated for a further 7 days under the same conditions, both GelMA microcarrier formulations supported cell expansion to approximately 12 000 cells per cm^2^ with no sign of cytotoxicity (Figure 2B). GelMA microcarriers are theoretically digestible, dismissing the need for separation. To test this possibility, microcarriers were incubated with commercially available cell detachment proteases. Microcarriers were completely degraded by 0.25% trypsin/0.1% EDTA, 0.025% trypsin/0.01% EDTA, 0.05, or 0.005% TrypZEAN after 5 minutes, while full‐strength Accutase did not degrade the microcarriers even after 15 minutes (Figure 2C).

### 3.3 Expansion of ihMSCs in RWVBs

**FIGURE 3:**
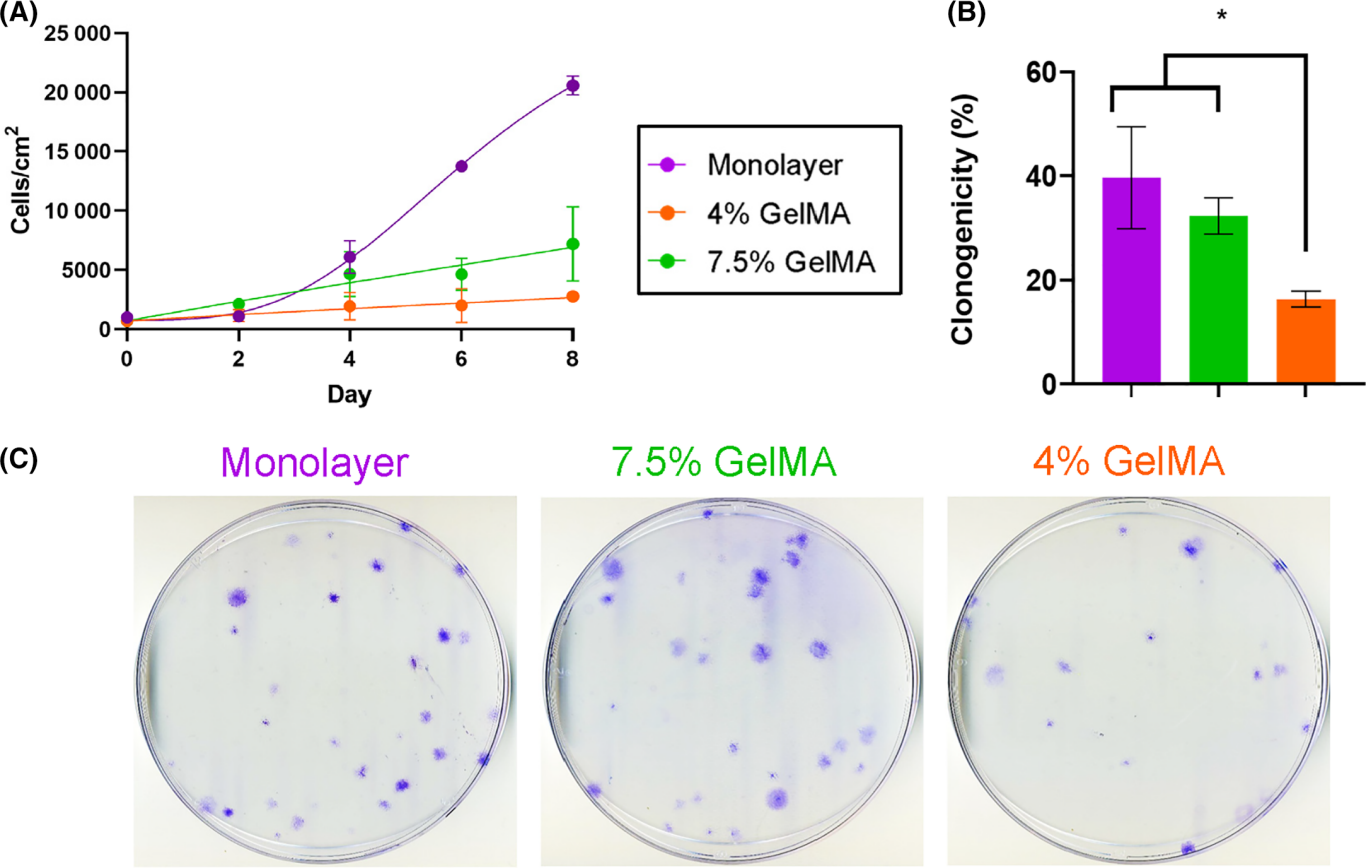
Expansion of ihMSCs on microcarriers in RWVBs: A, Growth curves of ihMSCs cultured in RWVBs. B, Quantification of colony forming potential of ihMSCs recovered from monolayer cultures, 4% GelMA microcarriers or 7.5% GelMA microcarriers. Statistics: n = 3, one‐way ANOVA with Tukey post‐test, *P < .05. Representative images of colony forming unit assays are shown in C

Initial bioreactor cultures were conducted in RWVBs with 4% and 7.5% GelMA microcarriers. Cells attached to 7.5% GelMA microcarriers proliferated more slowly than cells in monolayer, but reproducibly generated final densities of approximately 8000 cells per cm^2^ after 8 days of culture (Figure 3A). Cells grown on 4% GelMA in RWVBs had significantly reduced proliferative capacity with a final density of approximately 2000 cells per cm^2^ after 8 days (Figure 3A); an observation that was surprising given equivalent yields on plated cultures (Figure 2B). When ihMSCs were recovered by microcarrier degradation and subjected to standard assays of single‐cell derived clonogenicity, 7.5% GelMA maintained colony forming unit (CFU) potential comparable to monolayer culture, but cells grown on 4% GelMA had markedly reduced CFU potential (Figure 3C). The effects of growth on 4% GelMA therefore appeared to persist after plating on monolayers and affected the establishment of single‐cell derived colonies.

**FIGURE 4:**
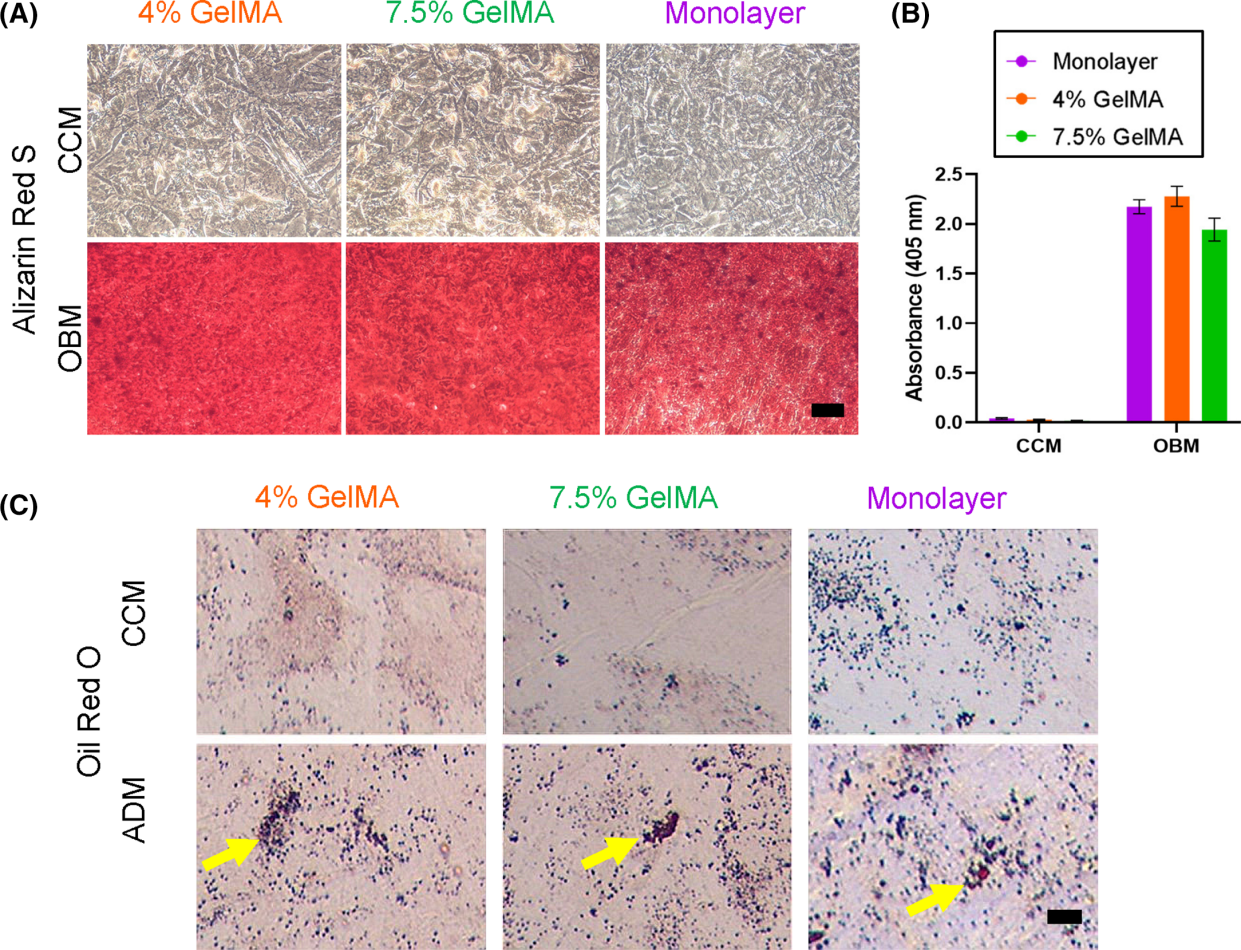
Osteogenic and adipogenic differentiation potential after RWVB expansion on GelMA microcarriers: A, Representative images of ARS stained monolayers after 21 days of culture in the osteogenic basal media (OBM) or complete culture media (CCM) (bar = 300 μm). B, ARS extraction and spectrophotometry demonstrate there was no difference in ARS staining between mineralized monolayers. Statistics: n = 3, one‐way ANOVA with Tukey post‐test, all *P* values were above .05. C, Representative images after 28 days of culture in adipogenic differentiation media (ADM) (scale bar = 200 μm). Small, sparsely populated oil red O‐stainable vacuoles are arrowed

The ihMSCs were then plated at high density into monolayers and subjected to standard assays of osteogenic and adipogenic potential. Both ihMSCs cultured on 7.5% and 4% GelMA generated an inducible mineralized monolayer that could be stained with the calcium binding dye ARS (Figure 4A). When the ARS was recovered and quantified, the degree of staining was equivalent between the GelMA microcarrier ihMSCs and monolayer expanded controls (Figure 4B). The ihMSCs were also subjected to adipogenic conditions in high density monolayer culture (Figure 4C). Consistent with previous reports by this group and others,^25^, ^36^, ^41^, ^42^, ^43^ ihMSCs from all 3 conditions exhibited a modest adipogenic response, with small, sparsely populated oil red O‐stainable vacuoles evident in all cases, but most apparent in cultures that originated from 7.5% GelMA or monolayers. In parallel experiments with primary human dermal fibroblasts, approximately 30‐fold expansion could be attained after 8 days in RWVs, and the cells could be recovered by trypsinization with no detectable cell death.

### 3.4 Expansion of ihMSCs in VWBs

**FIGURE 5:**
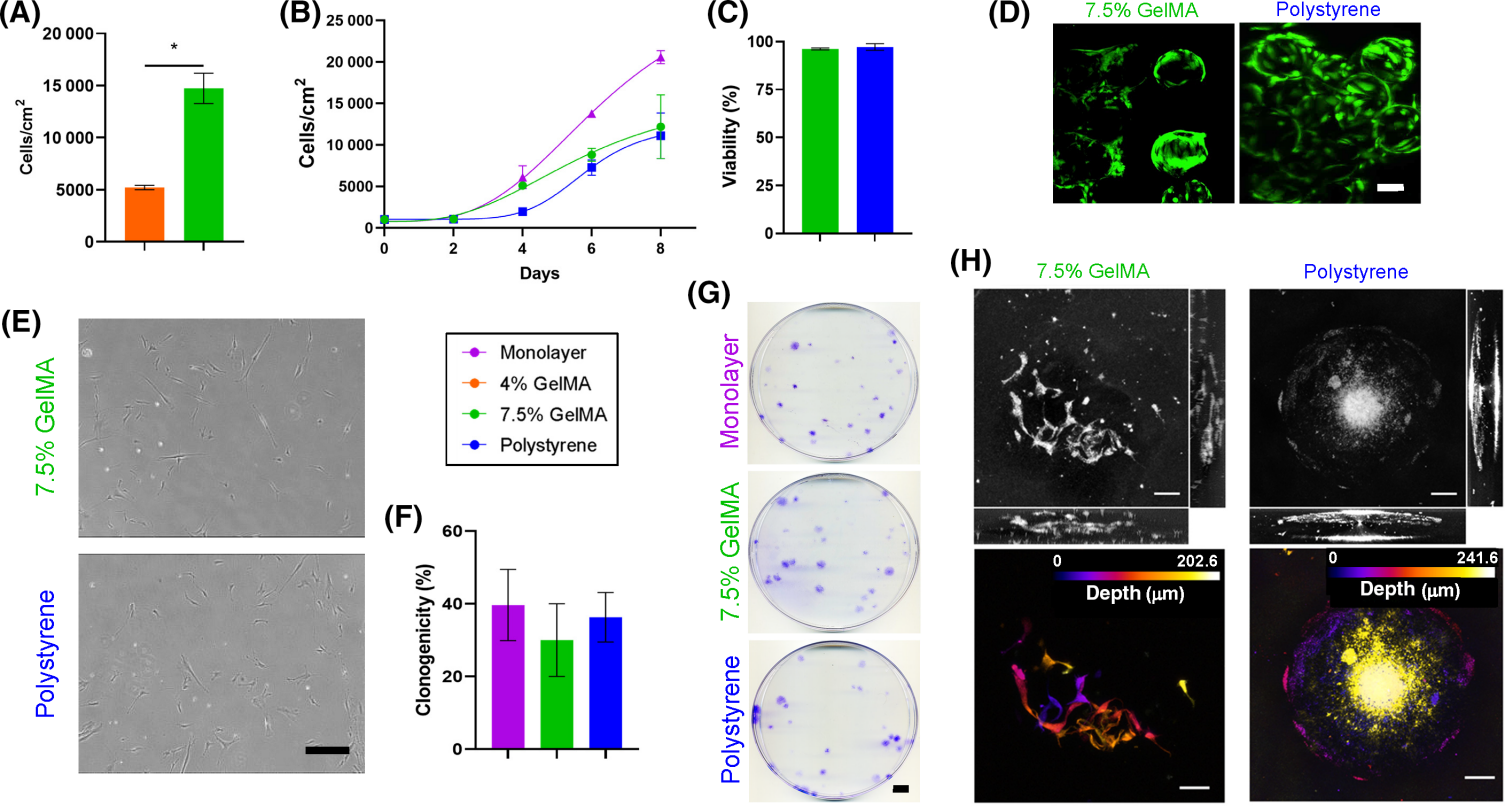
Expansion of ihMSCs on microcarriers in VWBs: A, Final yields after 8‐days of culture with comparison between 4% and 7.5% GelMA microcarriers. At least five test runs demonstrated that 4% GelMA microcarriers were inferior to 7.5% GelMA microcarriers for expanding numbers of ihMSCs. Statistics: n = 3, Student's *T* test, *P > .05. B, Growth curves of ihMSCs cultured in VWBs. Statistics: n = 4 to 5, differences were evaluated using a repeated‐measures ANOVA, with the means on each day being compared via Sidak's multiple comparisons test. Monolayer vs 7.5% GelMA at day 6 and 8 (*P* < .05), polystyrene vs GelMA 7.5% at day 4 (*P* < .05). C, Viability of ihMSCs recovered from VWBs. Comparable data were generated from three independent cultures. Statistics: n = 3, Student's *T* test, *P* > .05. D, Live/dead staining of ihMSC laden microcarriers after 4 days of culture in VWRs. Green cytoplasmic stain indicates live cells and red nuclei indicate dead or dying cells. No evidence of infiltration was apparent. Scale bar = 100 μm. E, Monolayer culture of recovered ihMSCs indicates that the typical spindle‐shaped morphology is retained (scale bar = 100 μm). F, Quantification of colony forming potential of ihMSCs recovered from monolayer cultures, 7.5% GelMA microcarriers or polystyrene microcarriers. Statistics: n = 3, one‐way ANOVA with Tukey post‐test, *P* > .05. G, Representative images of colony forming unit assays (scale bar = 100 cm). H, Two‐dimensional Z‐projections of ihMSCs on 7.5% GelMA (left) and polystyrene (right) microcarriers. Confocal volumes of ihMSCs on microcarriers imaged in reflectance mode (above). Depth‐color coded Z‐projection (below) of ihMSCs demonstrating that GelMA microcarriers permit imaging much deeper into the microcarriers than with polystyrene. Scale bar = 50 μm. Voxel size in original image: 0.2836 × 0.2836 × 2.4118 μm^3^

To determine whether GelMA microcarriers shared the same growth limitations as observed in RWVBs, 8‐day trials were performed with microcarriers seeded with 1000 ihMSCs per cm^2^ in 100 mL capacity VWBs. In these experiments, the 7.5% GelMA microcarriers generated approximately 13 000 cells per cm^2^ after 8 days, and while this was lower than what is generally observed on monolayers, 10 000 to 15 000 cells per cm^2^ is deemed an appropriate density at harvest given that extended duration at confluence can be a detriment to downstream applications.^32^, ^44^, ^45^ Three‐fold fewer ihMSCs were consistently recovered from 4% GelMA microcarriers than 7.5% GelMA microcarriers (Figure 5A) and as a result, only 7.5% GelMA microcarriers were used for subsequent VWB experiments.

From growth curve assays, the expansion rate of ihMSCs was similar on polystyrene and 7.5% GelMA microcarriers (Figure 5B), with comparable viability (Figure 5C). Cell recoveries at day 8 were comparable to the data presented in Figure 5A, yielding between 8800 and 16 300 cells per cm^2^ with a mean density of 12 667 cells per cm^2^. Millimeter‐sized microcarrier aggregates were observed on days 6 and 8 of expansion on 7.5% GelMA microcarriers and longer duration in culture did not result in increased recoveries. Live/dead staining indicated no dying cells on GelMA microcarriers, in the aggregates or on polystyrene microcarriers (Figure 5D). To explore the potential for linear scale‐up, a preliminary expansion was performed on 7.5% GelMA using 500 mL VWB systems resulting in a yield of 1.7 × 10^7^ cells at a density of 14 448 cells per cm^2^ at 98% viability after harvest.

After isolation from the microcarriers, the identity of ihMSCs was verified according to the International Society for Cell Therapy criteria, requiring that in addition to multi‐lineage differentiation, the cells generate single‐cell derived colonies, adopt a spindle‐shaped fibroblastoid morphology and possess the expected surface immunophenotype.^46^ Following bioreactor expansion, cells were recovered by trypsinization and plated onto tissue culture plastic. The ihMSCs adopted a spindle‐shaped morphology with a majority having a central halo on phase contrast microscopy, features typical of hMSCs (Figure 5E). These features were present regardless of whether cells were cultured on 7.5% GelMA or polystyrene. Furthermore, the CFU potential of ihMSCs expanded on 7.5% GelMA or polystyrene microcarriers were not significantly different than that of monolayer‐expanded ihMSCs (Figure 5F,G).

To examine whether ihMSCs could be imaged on the microcarriers without need for labeling or staining, immobilized cell‐laden microcarriers were subjected to confocal microscopy in reflectance/scattering mode. In the case of GelMA microcarriers, cell bodies were clearly visible on the microcarriers (Figure 5H, above left) at a depth range of 2.41 to 202.6 μm which encompassed the entire depth of the sphere (Figure 5H, below left). In the case of the polystyrene microcarriers, imaging was significantly perturbed by significant scattering and a focusing effect that washed out the majority of the image data (Figure 5H, above right). While some morphological data could be collected from the periphery of the spheres, the depth range was limited to approximately 2.4 to 100 μm (Figure 5H, below right).

**FIGURE 6:**
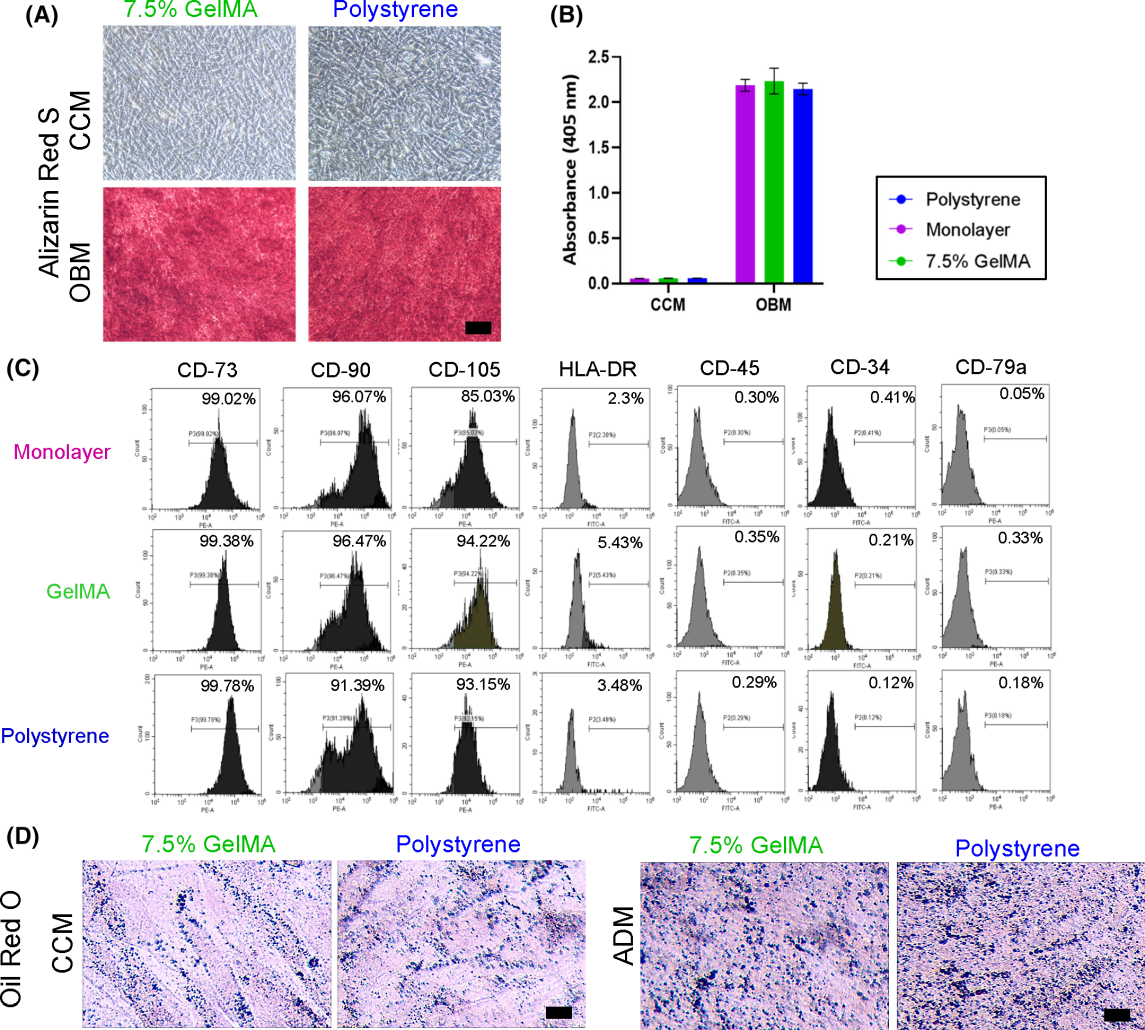
Osteogenic and adipogenic differentiation potential after VWB expansion on GelMA microcarriers: A, Representative images of ARS stained monolayers after 21 days of culture in the osteogenic basal media (OBM) or complete culture media (CCM) (scale bar = 300 μm). B, ARS extraction and spectrophotometry demonstrate there was no difference in ARS staining between mineralized monolayers. Statistics: n = 3, one‐way ANOVA with Tukey post‐test, all *P* values were above .05. C, Histograms of ihMSCs stained with indicated surface antigens after being cultured on collagen‐polystyrene or 7.5% GelMA microcarriers in 100 mL VWBs for 8 days with comparison to monolayer cultures. Positive gating is indicated by horizontal line. D, Representative images after 28 days of culture in adipogenic differentiation media (ADM) (scale bar = 200 μm)

The osteogenic differentiation potential of ihMSCs was maintained after microcarrier expansion in VWBs, with dense ARS staining of mineralized monolayers (Figure 6A,B). As expected, response to adipogenic stimulus was modest but detectable, with ADM inducing the formation of sparsely distributed fat‐filled droplets that were stainable with oil red O (Figure 6D). The osteogenic and adipogenic differentiation observed in the VWBs was similar to differentiation observed after RWVB expansion on 4% and 7.5% GelMA microcarriers and cells grown on monolayers.

Flow cytometry of cells cultured on monolayers, 7.5% GelMA microcarriers and polystyrene carriers all showed expression of CD‐73 and CD‐105 while being negative for CD‐11b, CD‐14, CD‐19, CD‐34, CD‐45, CD‐79a, and HLA‐DR (Figure 6C, Supporting Information S3A), indicating that neither microcarrier causes a change in the immunophenotype of ihMSCs. Reverse transcriptase real‐time PCR assays for pluripotency markers *Nanog*, *Oct4*, *Sox2*, *Utf1* and hTERT demonstrated that GelMA culture caused a slight increase in the transcription of these genes, but given that the source iPS cells expressed several thousand fold higher levels of transcript,^24^ the physiological significance of these results is likely to be marginal. The same was true for mesodermal biomarker CD140A, ectodermal biomarker E‐cadherin (*Ecad*) and endodermal biomarker *Foxa2* (Supporting Information S3B).

### 3.5 Immunomodulatory potential of ihMSCs expanded on microcarriers in RWVBs and VWBs

**FIGURE 7:**
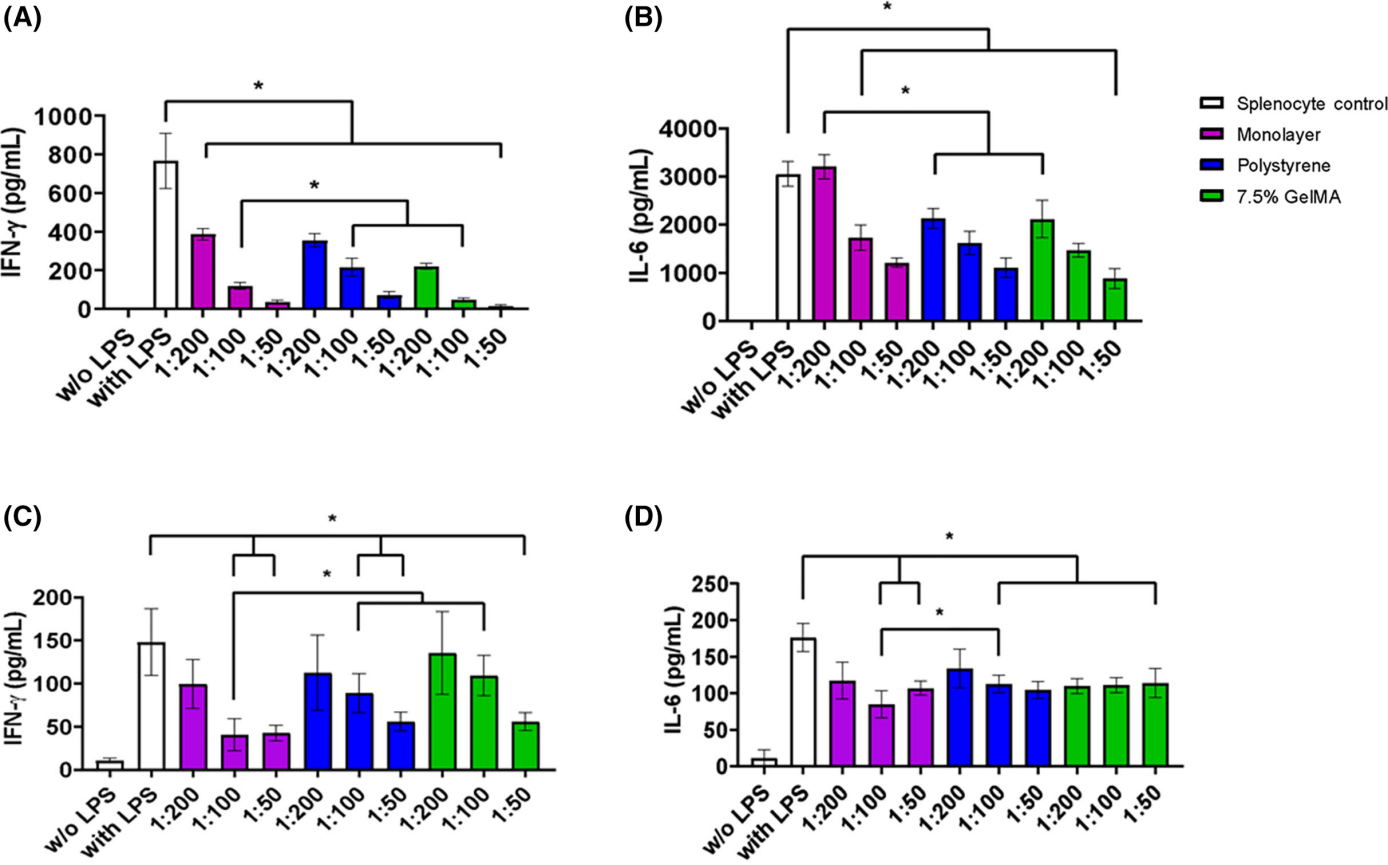
GelMA microcarrier expansion in RWVBs or VWBs maintains in vitro immunomodulatory capabilities of ihMSCs. A,C, Quantification of IFN‐γ release over 24 hours by LPS stimulated murine splenocytes in presence of ihMSCs from 7.5% GelMA microcarriers, collagen‐polystyrene microcarriers or monolayers. B,D, Quantification of IL‐6 release under same conditions. A,B, ihMSCs expanded in RWVBs compared with monolayer controls. C,D, ihMSCs expanded in VWBs compared with monolayer controls. Statistics: n = 6, one‐way ANOVA with Tukey post‐test, *P > .05

A series of in vitro experiments were performed to determine whether microcarrier expansion in bioreactors altered their immunomodulatory potential. The ihMSCs from each bioreactor were cocultured for 24 hours in the presence of LPS‐activated BALB/c murine splenocytes. Under control conditions, splenocytes drastically upregulate secretion of inflammatory cytokines such as IFN‐γ and IL‐6 after challenge with LPS. Immunomodulatory ihMSCs would be expected to blunt this response in a dose‐dependent manner. Cells isolated from 7.5% GelMA and polystyrene microcarriers cultured in RWVBs were able to inhibit output of IFN‐γ and IL‐6 in a dose‐dependent manner to a degree equivalent to monolayer‐expanded cells (Figure 7A,B). In the case of IFN‐γ, the same was true for ihMSCs isolated in VWBs (Figure 7C). In the case of IL‐6, secretion was blunted at all cell doses tested, but a dose response relationship was not apparent in that all doses reduced IL‐6 output by 30% to 50% (Figure 7D). The reason for a lack of dose‐dependency is unclear, but the overall upregulation of IFN and IL‐6 were less prominent in this set of assays, potentially affecting the ability to detect a dose dependence.

## 4 DISCUSSION

In this study, we demonstrated that GelMA microcarriers support the expansion of ihMSCs in RWVBs and VWBs without detriment to clonogenicity, differentiation potential or immunomodulatory capacity. The microcarriers described here have noteworthy advantages when compared with competing products: (a) the microcarriers can be generated using a cost‐effective and scalable manufacturing strategy, (b) their reproducible size and optical properties improve compatibility with noninvasive imaging techniques and reproducibility of 3D image acquisition, and (c) isolation of ihMSCs after bioreactor expansion on GelMA microcarriers is streamlined through direct dissolution of the microcarriers, thus avoiding a cell separation step and associated cell losses. After GelMA microcarrier culture, ihMSCs showed at least comparable, if not greater, immunomodulatory potential than monolayer‐cultured cells.

Porcine gelatin products generated from traceable tissue sources are in widespread use (eg, Gelfoam, plasma substitutes, hemostats, femoral plugs). While GelMA hydrogels are not currently utilized in patients, highly purified GelMA with batch‐reproducible characteristics has been reported, utilizing a mixed‐batch one‐pot strategy.^47^ The gelatin used in this study was purchased sterile and handled aseptically, but preliminary studies confirmed that GelMA beads could be E‐beam sterilized if necessary. In terms of reproducibility, multiple batches of GelMA microcarriers were generated from different batches of gelatin, and all generated microcarriers of comparable size and had the capacity to expand ihMSCs within expected parameters.

The use of step emulsification microfluidic devices for the generation of microcarrier batches is appealing due to their ability to maintain low variations in droplet diameter despite variations in flowrate.^40^, ^48^ The 100‐channel device used in this study was able to produce uniform 120 μm microcarriers over 2 hours of continuous production, similar to observations in previous publications.^48^ In terms of yields and rate of manufacture, the maximum operating flow rates in step emulsification microfluidics are directly proportional to the number of channels and can be scaled in parallel. For example, 100 channels would be predicted to generate the equivalent of 10 000 cm^2^ surface area within 4 hours of operation which would be equivalent to operation of a 2 L bioreactor. Two hundred channels in the unit would reduce the time parameter to 2 hours and so on.^40^ To remove oil, microcarriers were subjected to centrifugation while floating on a bed of glycerol. At the current scale, a 20 mL glycerol bed cleaned sufficient microcarriers for 10 100‐mL cultures. Using laboratory‐scale centrifugation equipment, this process could be scaled up significantly. For example, a modest centrifuge processing four 1‐L vessels could generate microcarriers sufficient for a 40‐L culture in less than 5 minutes.

Considering the raw materials required, we estimated a cost of $0.010 per cm^2^ to make GelMA microcarriers, comparable to the retail cost of plastic microcarriers at approximately $0.030 per cm^2^. Recently, Ng et al reported a single‐channel droplet‐fluidics device with the capacity to generate 180 μm diameter genipin‐crosslinked gelatin spheres.^49^ The rate of sphere production in the Ng study required 10 hours of run time to supply a 100 mL culture which is 50 times slower than what was achieved with our 100 channel system. Furthermore, genipin crosslinking of gelatin took 2 days to complete compared with seconds for photocrosslinking GelMA.

The main benefits of RWVBs and VWBs are low fluid shear forces which avoids detrimental effects to hMSC expansion in stirred bioreactors^50^, ^51^, ^52^, ^53^ and reduces the potential for physical damage to the GelMA. RWVBs minimize shear forces by causing the cell culture media and microcarriers to rotate as a fluid mass though rotational acceleration of the entire reactor.^54^ The impeller design in the VWB homogenizes fluid and hydrodynamic forces by mixing in both axial and radial directions simultaneously avoiding turbulent mixing seen in stirred tank and rocking bag reactors.^51^

We originally pursued the use of 4% GelMA microcarriers for cell expansion as previous studies have demonstrated cell expansion in well plates after cell encapsulation in 4% to 5% GelMA hydrogels.^55^, ^56^, ^57^ In early experiments utilizing plated microcarrier cultures, yields of ihMSCs from microcarriers made of 7.5% and 4% GelMA formulations resulted in comparable yields of cells. However, the translation of 4% GelMA from low‐attachment plates to bioreactors led to a 3‐fold decrease in the number of recovered cells after normalization to culture area. Given that the plated cultures yielded comparable results, the likelihood that the stiffness of the substrate alone contributed to the reduced yields is unlikely. While both bioreactors cause the cell‐laden microcarriers to be suspended through motion of the liquid media, the combined effect of suspension culture and soft substrate is also unlikely to account for the reduced effectiveness of 4% GelMA given that RWVBs exhibit negligible shear stress on cells.^58^ At present, the reason for reduced proliferation of ihMSCs on 4% GelMA is unclear, but combined effects of substrate stiffness, suspension in culture and also the geometry of microcarriers have been reported to result in unpredictable results in similar studies utilizing tissue derived hMSCs.^59^

The stiffness of attachment substrate can influence the differentiation potential and proliferation rate of bone marrow derived MSCs.^60^, ^61^ Engler et al reported that GelMA substrates of stiffness 1, 10, and 25 to 40 kPa was optimal for activation of neural, myogenic, and osteogenic biomarkers, respectively.^60^ Xu et al reported that umbilical cord derived MSCs proliferated more rapidly on GelMA with a stiffness of 13 to 16 kPa and the rate declined slightly as stiffness increased. The Xu study did not assay gels with lower stiffness, but given the low proliferative activity on 4% GelMA in this study, it appears that the optimal window for proliferation of MSCs on GelMA could be in the region of 10 to 16 kPa. The mean compressive moduli (an estimate of stiffness) of 4% and 7.5% GelMA materials were 1 and 7.9 kPa, respectively. Differentiation assays performed on polystyrene plates using cells expanded on 4% GelMA or 7.5% GelMA indicated comparable osteogenic or adipogenic differentiation as with cells that were expanded on polystyrene plates. In agreement with other studies,^43^, ^62^, ^63^, ^64^, ^65^ ihMSCs exhibited limited adipogenic capacity, indicating that this was an inherent characteristic of the cells rather than growth conditions. Together, these results indicate that the ihMSCs remain undifferentiated during expansion on GelMA microcarriers.

A desirable characteristic of microcarriers is the potential for rapid imaging without need for invasive labeling procedures. The GelMA spheres permitted imaging using reflectance confocal microscopy throughout the entire depth of the spheres resulting in clearly discernable morphology of the cell bodies based solely on endogenous scattering of cells. In contrast, polystyrene microcarriers caused extensive background scattering and a focusing effect that perturbed imaging of cells located at the proximal or distal surface. While some morphological data could be acquired, the data were partial and subject to a poor signal to noise ratio. The reason for this effect is in part due to polystyrene possessing a much higher refractive index (1.59) compared with GelMA (1.35). In these experiments, the microcarriers were immobilized in agarose because standard volumetric microscopy takes seconds to perform. Rapid imaging of multiple microcarriers using high‐speed confocal or light‐sheet microscopy is feasible without need for agarose immobilization, but this is best achieved on microcarriers with a narrow size distribution. The GelMA microcarriers described herein possess an average diameter of 120 μm and a deviation of 20 μm (approximately 17%). In contrast, the diameters of commonly utilized polystyrene microspheres can frequently deviate by 25% to 30%.

An attractive attribute of the GelMA microcarriers is the potential for their complete degradation within 5 minutes with standard cell dissolution reagents, facilitating the release of cells without need for separation from the microcarriers resuting in over 95% viability. Commercial degradable microcarriers are available, but their porosity can facilitate infiltration of hMSCs resulting in unpredictable growth characteristics^20^, ^21^ and it has been recently reported that extended growth of hMSCs on porous gelatin can result in extracellular matrix deposition that precludes timely degradation of the microcarriers to release cells.^66^ A potential concern posed by the use of degradable GelMA microcarriers is the release of degradation products that might be toxic to cells, but the duration of this exposure is short (5 min) effectively dismissing the probability of internalization. Degradation products released by digestion of GelMA expected to largely consist of peptide segments with intact methacrylate crosslinks since trypsin targets peptide bonds in the gelatin backbone of GelMA. Some free methacrylate, methacrylamide, and methacrylic acid may also be present that have the capacity to kill cells at concentrations between 2.5 and 25 mg mL^−1^, but this is significantly higher than is possible during dissociation of ihMSCs from the GelMA microcarriers.^67^ While there is some concern that these trace compounds may contribute to malignancy, poly‐methyl methacrylate glues frequently used orthopedic applications exposes patients to higher levels of degradation products, and this has not been linked to increased cancer risk over decades of use.^68^ It is also noted that exposure of ihMSCs to FBS during culture is associated with risk of immune reaction to bovine antigens^69^ when infused into patients, but preliminary data generated by this group indicates that ihMSCs can be expanded on GelMA microcarriers in the presence of 5% (v/v) human platelet rich plasma for 8 days (n = 2, 12.4‐fold, SD 3.9 and 28.2‐fold, SD 3.4) and future work is directed at optimizing these parameters.

While the degree of expansion in RWVBs was relatively low (approximately 8‐fold over 8 days), the rate of growth of ihMSCs on 7.5% GelMA microcarriers in VWBs was comparable to polystyrene beads, with viable yields of 8800 to 16 300 cells per cm^2^ attainable after 8 days. This is comparable to 12‐13‐fold expansion previously reported for bone marrow derived hMSCs expanded in a 2.2 L VWBs on Synthemax II beads over a 14 day period^51^ and in a 0.1 L VWBs on plastic beads over a 7 day period.^12^ Expansion levels were also superior to those reported by Ng et al (5‐6‐fold) on gelatin spheres using a 125 mL spinner flask over 10 days. Interestingly, with the growth media employed, the cell density remained capped at approximately 16 000 cells per cm^2^ even after extended durations on microcarriers that would normally lead to confluence on monolayers. While this indicates that there are some limitations to yield from microcarriers, the density threshold observed is within the parameters reported to preserve the RS phenotype associated with optimized functionality and favorable therapeutic characteristics of MSCs.^32^, ^44^, ^45^ Indeed, when ihMSCs recovered from GelMA spheres were replated on standard tissue culture plastic, they adopted a spindle‐shaped morphology associated with the RS phenotype. The type of VWB used in this study was the PBS Biotech Bioreactor System with pilot studies primarily performed on the 100 mL system. We were able to employ the 500 mL system to generate a density of cells at harvest equivalent to 100 mL cultures indicating the capacity for linear scale‐up. PBS bioreactors are scalable to up to 500 L with current models to accommodate 3, 15, and 80 L.

The density of spheres used in these experiments was designed to mimic the media volume: growth area ratio used in standard monolayer cultures, which was 5 cm^2^ per mL of media. Further work is necessary to elucidate the effects of decreasing the media volume: growth area ratio and the duration of culture with a view to increasing potential yields, especially in RWVBs. This should be regarded with caution, however, given the potential to form aggregates in the latter days of culture.

When ihMSCs were co‐incubated with murine splenocytes that were pre‐activated with LPS, a dose‐dependent reduction in the secretion of inflammatory cytokines was observed, indicating that the bioreactor‐generated ihMSCs have the theoretical capacity to blunt early‐stage inflammatory responses triggered by the engagement of toll‐like receptor 4 (TLR4)^70^ and therefore may have the capacity to modulate inflammatory responses in vivo in a similar manner to tissue‐derived hMSCs.^71^, ^72^ Bioreactor‐expanded sources of tissue‐derived hMSCs have been reported to blunt inflammatory responses in in vitro assays,^73^, ^74^ and the immunomodulatory capacity of iPSC‐derived hMSCs has been demonstrated,^25^ but to our knowledge, this is the first time immunomodulatory ihMSCs have been generated using a scalable VWB platform.

Herein, we demonstrate an approach for the scalable generation of iPS‐derived hMSCs that show promise for repair of skeletal tissues and immune modulation, but the prospect of expansion of iPS cells using GelMA microcarriers is also raised by this study. Previous reports indicate that iPS cells can be successfully expanded while attached to various forms of coated microcarrier in stirred‐tanks or spinner flasks but the coating, mode of agitation, and the size of aggregates that invariably form can affect yields, viability and functionality.^75^ Detachment of the cells from the microcarriers, dissociation to single cells, and filtration processes can further affect viability and functionality.^75^ To dismiss the complications associated with microcarriers, iPS cells have also been expanded as free‐floating cellular aggregates in PBS‐VWB systems.^76^, ^77^ Indeed, the body of work on the culture of iPS cells in VWB systems is primarily focused on expansion in the aggregate form, but given that GelMA microcarriers are soft, less likely to cause cell damage and are rapidly degraded by light enzymatic treatment, GelMA microcarriers may be well‐suited to large scale expansion of iPS cells and well as their iPS‐derived MSC progeny.

## 5 CONCLUSION

A scalable method for expansion and rapid harvest of iPS‐derived hMSCs is described based on attachment to custom‐synthesized gelatin‐methacryoyl microcarriers and culture in vertical wheel bioreactors. The system permits approximately 16‐fold expansion of cells in 8 days, facilitates rapid harvest by digestion of the microcarriers, and exhibits superior optical properties for cell visualization when compared to polystyrene microcarriers. Recovered cells maintain differentiation and immune modulation capacity.

## CONFLICT OF INTEREST

F.L. declared patent ownership, “Mesenchymal stem cells derived from induced pluripotent stem cells,” which depicts some of the concepts presented herein and research funding from DoD CDMRP PCRP PC150083 and NIH R21DE027457. The patent was granted in the United States on 2019‐07‐16 (US10351825B2) and is pending internationally (WO2016081032A3). The other authors declared no potential conflicts of interest.

## AUTHOR CONTRIBUTIONS

R.E.R.: conceptualized the research, performed the experiments, interpreted the data, wrote the manuscript, proofed and approved manuscript; A. Haskell: conceptualized the research, performed the experiments, interpreted the data, wrote the manuscript, proofed and approved manuscript; B.P.W.: performed experiments, proofed and approved manuscript; S.D., M.L., D.T., S.P., G.K., H.K., H.B.: performed experiments, proofed and approved manuscript; S.L.W.: performed experiments, conceptualized research, awarded financial support to perform the work, proofed and approved manuscript; O.R.B., J.D., Q.Z.: performed experiments, designed and synthesized microfluidic unit, proofed and approved manuscript; K.C.M.: conceptualized the research, performed the experiments, interpreted the data, wrote the manuscript, proofed and approved manuscript; A. Han: designed and synthesized microfluidic unit, proofed and approved manuscript; Z.L.N. and R.L.: conceptualized research, awarded financial support to perform the work, proofed and approved manuscript; F.L.: conceptualized the research, performed the experiments, interpreted the data, wrote the manuscript, performed experiments, proofed and approved manuscript; C.A.G. and R.K.: conceptualized the research, performed the experiments, interpreted the data, wrote the manuscript, conceptualized research, awarded financial support to perform the work, proofed and approved manuscript.

## Supporting information


**Figure S1**. Summary of experimental plan.

**Figure S2**. Video demonstration of step emulsification microfluidic device producing 4% GelMA microcarriers in real time: The GelMA header (top) dead‐ends on the right while the oil channel (bottom) continues to the outlet port of the microfluidic device, forcing the GelMA solution downward through the channels forming GelMA droplets in the oil channel. The outlet of the oil channel is passed through a UV source for crosslinking before ending in a collection tube.

**Figure S3**. The immunophenotypes of VWB‐expanded ihMSCs are comparable to monolayer cultures. Panel a: Histograms of ihMSCs stained with FITC (CD‐34, CD‐45, HLA‐DR, CD‐90) or PE (CD‐11b, CD‐14, CD‐19, CD‐73, CD‐79a, CD‐105) after being cultured on collagen‐polystyrene or 7.5% GelMA microcarriers in 100 mL VWBs for 8 days with comparison to monolayer cultures. Positive gating is indicated by horizontal line. Panel b: RT‐PCR assays for pluripotency‐associated genes (grey bars) or biomarkers for ectoderm (*Ecad*), endoderm (*Foxa2*) or mesoderm (*CD140A*).

## Data Availability

The data that support the findings of this study are openly available in the Aggie Cloud Server at [URL pending. Raw data can be requested directly from corresponding authors].
