# Supplementary figures and images for: A scalable system for generation of mesenchymal stem cells derived from induced pluripotent cells employing bioreactors and degradable microcarriers
Source: Stem Cells Transl Med. 2021 Sep 10;10(12):1650–65. doi: 10.1002/sctm.21-0151 (PMC8641084; doi:10.1002/sctm.21-0151)

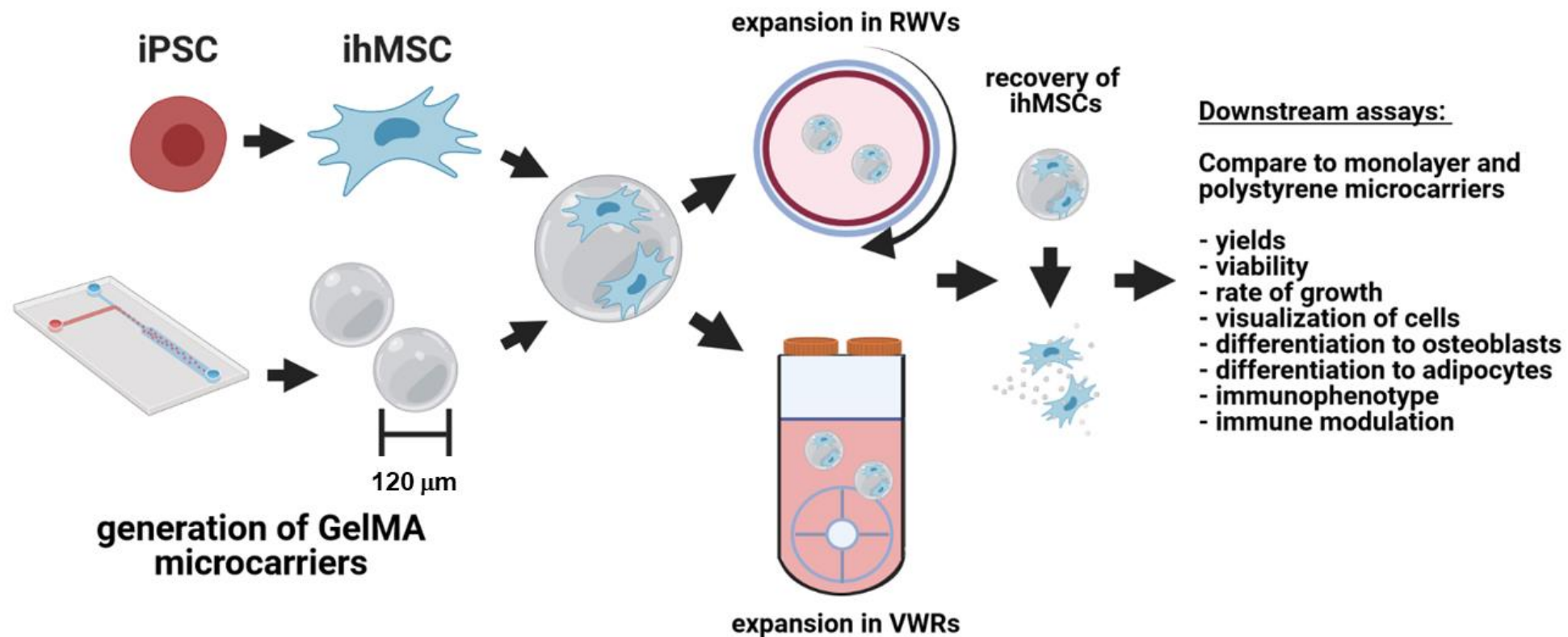

Supplement: Supplementary file 1 — Figure S1. Summary of experimental plan. [file SCT3-10-1650-s003.pdf]

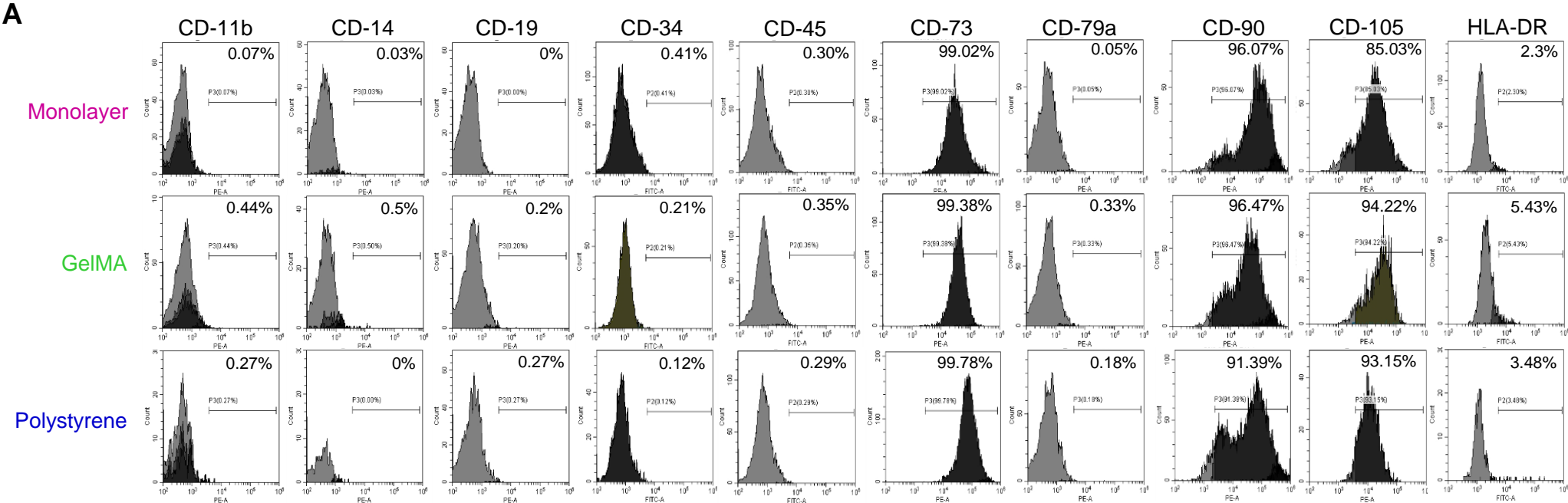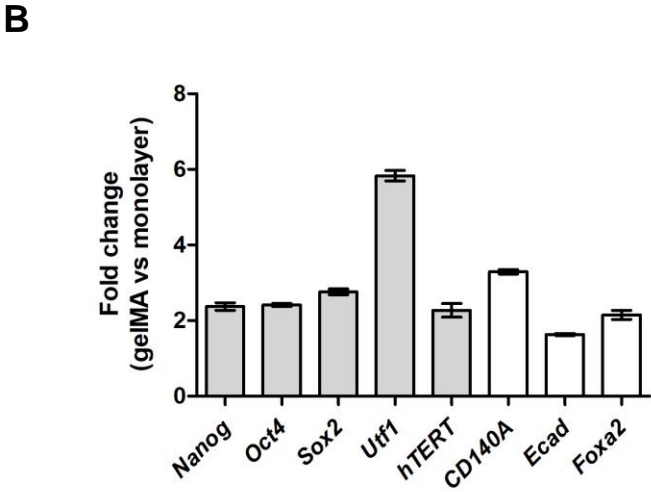

Supplement: Supplementary file 3 — Figure S3. The immunophenotypes of VWB‐expanded ihMSCs are comparable to monolayer cultures. Panel a: Histograms of ihMSCs stained with FITC (CD‐34, CD‐45, HLA‐DR, CD‐90) or PE (CD‐11b, CD‐14, CD‐19, CD‐73, CD‐79a, CD‐105) after being cultured on collagen‐polystyrene or 7.5% GelMA microcarriers in 100 mL VWBs for 8 days with comparison to monolayer cultures. Positive gating is indicated by horizontal line. Panel b: RT‐PCR assays for pluripotency‐associated genes (grey bars) or biomarkers for ectoderm (Ecad), endoderm (Foxa2) or mesoderm (CD140A). [file SCT3-10-1650-s001.pdf]
